# Global health leadership training in resource-limited settings: a collaborative approach by academic institutions and local health care programs in Uganda

**DOI:** 10.1186/s12960-015-0087-2

**Published:** 2015-11-18

**Authors:** Damalie Nakanjako, Elizabeth Namagala, Aggrey Semeere, Joanitor Kigozi, Joseph Sempa, John Bosco Ddamulira, Achilles Katamba, Sam Biraro, Sarah Naikoba, Yohana Mashalla, Carey Farquhar, Afya Bora Consortium members, Nelson Sewankambo

**Affiliations:** Department of Medicine, Makerere University College of Health Sciences, P.O. Box 7072, Kampala, Uganda; Ministry of Health, AIDS Control Program, Kampala, Uganda; Infectious Diseases Institute, Makerere University College of Health Sciences, Kampala, Uganda; Medical Research Council/Uganda Virus Research Institute, Entebbe, Uganda; Departments of Medicine, Global Health and Epidemiology, University of Washington, Seattle, WA USA; University of Botswana, Gaberone, Botswana

**Keywords:** Global health, Health leadership, Training, Resource-limited settings, Sub-Saharan Africa, Uganda, Collaboration

## Abstract

**Introduction:**

Due to a limited health workforce, many health care providers in Africa must take on health leadership roles with minimal formal training in leadership. Hence, the need to equip health care providers with practical skills required to lead high-impact health care programs. In Uganda, the Afya Bora Global Health Leadership Fellowship is implemented through the Makerere University College of Health Sciences (MakCHS) and her partner institutions. Lessons learned from the program, presented in this paper, may guide development of in-service training opportunities to enhance leadership skills of health workers in resource-limited settings.

**Methods:**

The Afya Bora Consortium, a consortium of four African and four U.S. academic institutions, offers 1-year global health leadership-training opportunities for nurses and doctors. Applications are received and vetted internationally by members of the consortium institutions in Botswana, Kenya, Tanzania, Uganda, and the USA. Fellows have 3 months of didactic modules and 9 months of mentored field attachment with 80% time dedicated to fellowship activities. Fellows’ projects and experiences, documented during weekly mentor-fellow meetings and monthly mentoring team meetings, were compiled and analyzed manually using pre-determined themes to assess the effect of the program on fellows’ daily leadership opportunities.

**Results:**

Between January 2011 and January 2015, 15 Ugandan fellows (nine doctors and six nurses) participated in the program. Each fellow received 8 weeks of didactic modules held at one of the African partner institutions and three online modules to enhance fellows’ foundation in leadership, communication, monitoring and evaluation, health informatics, research methodology, grant writing, implementation science, and responsible conduct of research. In addition, fellows embarked on innovative projects that covered a wide spectrum of global health challenges including critical analysis of policy formulation and review processes, bottlenecks in implementation of national HIV early infant diagnosis and prevention of mother-to-child HIV-transmission programs, and use of routine laboratory data about antibiotic resistance to guide updates of essential drug lists.

**Conclusion:**

In-service leadership training was feasible, with ensured protected time for fellows to generate evidence-based solutions to challenges within their work environment. With structured mentorship, collaborative activities at academic institutions and local health care programs equipped health care providers with leadership skills.

**Electronic supplementary material:**

The online version of this article (doi:10.1186/s12960-015-0087-2) contains supplementary material, which is available to authorized users.

## Introduction

There is a limited health workforce in sub-Saharan Africa, and many health care providers take on significant health leadership roles, despite minimal formal training in leadership skills. Results from a survey among health workers in Benin and Kenya showed that health facility heads (nurses and doctors) were unable to inspire health care delivery teams due to limited skills to provide performance-based appraisals and feedback to subordinates, among other non-financial motivators for high-performance teams [1]. Innovative on-the-job leadership-training programs are therefore required to equip health care providers with practical skills required to lead high-impact health care programs. In-country strategies to increase the capacity of the trained health workforce in developing countries require additional expertise from local and international partners in health care [2,3]. To respond to the increased need of innovative experiential leadership training for nurses and doctors in an African setting, the Afya Bora Global Health Leadership training program was initiated in 2011 through a consortium of four African and four American universities [4]. In-country experiences from the implementation of the program are yet to be documented by the consortium member countries. In Uganda, the program is implemented through the Makerere University College of Health Sciences (MakCHS) and her partner institutions, including the Ministry of Health (MOH) public health care programs, non-governmental institutions, and the President’s Emergency Plan For AIDS Relief (PEPFAR)-funded HIV/AIDS care programs that were used as field attachment sites for fellows’ practical experiences. The leadership-training intervention for health workers to improve health care delivery is based on the people-centered health systems’ strengthening framework which shows that the improved capacity to lead and manage improves effective use of resources and performance in service delivery [5]. In addition to infrastructure, tools, and systems, personal capacity for leadership and governance remains critical in the capacity-building pyramid for health system strengthening in developing countries [6]. In this paper, Makerere University’s experiences in equipping health care leaders with leadership skills through practical training and mentorship supported by mentors from both MakCHS and local health care facilities (used as attachment sites for the fellows’ 9-month practical leadership experience) are presented.

In this paper, we describe major experiences of the fellows, focusing on lessons learned from this experiential model of in-service global health leadership training, to guide further development of in-service training opportunities to enhance leadership skills of nurses and doctors in Uganda and other developing countries.

## Methods

### Program setting

The Afya Bora Consortium was founded in 2009 based on four pre-existing partnerships between US and African universities [4]. Between January 2011 and January 2015, fellows from the respective consortium member countries were recruited for the Afya Bora fellowship, as described by Farquhar and others [4]. A 1-year fellowship is advertised annually through the partner institutions, ministries of health, working group members, and alumni. Applications are received and vetted internationally by members of the consortium institutions in Botswana, Kenya, Tanzania, Uganda, and the USA. Using standardized interview questions and score sheets developed by the consortium working group, oral interviews are held in the respective countries and the best four candidates per country are selected as successful candidates. Fellows are excluded from interviews upon absence of letters to support institutional willingness to protect 80% of their time for fellowship activities during the 1-year fellowship period.

### Description of the curriculum

A competence-based curriculum was developed to provide African health care leaders with practical management and leadership skills to enhance leadership capacity for in-service health care providers in resource-limited settings. A year-long fellowship was designed to involve a total of 8 weeks of didactic lectures offered as three classroom learning blocks. The didactic modules were designed to equip fellows with skills in communication, leadership, monitoring and evaluation, implementation science, health informatics, research methods, grant writing, human resources, and budgeting, as well as global health policy and governance. The classroom-based modules employ case-based discussions in small groups and are draw on Africa-focused case studies. The 3-week residential blocks were separated by two 4.5-month long experiential trainings at identified local governmental and non-governmental organizations involved in health-related activities including the Ministries of Health in the four African countries. The curriculum included four additional online modules, namely responsible conduct of research, research methods, project management, and HIV/AIDS updates.

### Description of attachment sites

In-country academic, health care, and research institutions were visited by members of the working group and accredited as attachment sites for experiential leadership training. To qualify for accreditation, attachment institutions had to demonstrate availability of suitable infrastructure (e.g., office space to host the fellow and a variety of projects in which fellows could participate) as well as potential mentors to support fellows’ training. Attachment site rotations allow learners to apply concepts and skills learned in the classroom to real-world problems faced by local health organizations. Fellows had options of moving to attachment sites within their respective countries, and within other partner institutions in Africa. It was preferred that fellows move to attachment sites outside their working station, although fellows could also take on new “fellow roles” within their working stations.

### Mentorship at attachment sites

Fellows were encouraged to drive the mentorship processes at the attachment sites and to identify projects that would benefit both the attachment site and the fellow. Each fellow was assigned a primary mentor from the in-country working group and a site mentor who was by default a leader in the fellow’s area of interest at the selected attachment site. Mentoring training was provided by the working group at MakCHS, with mentor-fellow orientation workshops at the beginning, and monthly meetings through the 9-month attachment site period. The workshops were attended by primary mentors, attachment site mentors, and fellows. Fellows met at least weekly with attachment site mentors and often daily interactions occurred. Fellowship experiences and activities, documented during weekly mentor-fellow meetings and monthly mentoring team meetings, were compiled and analyzed manually using pre-determined themes to assess the effect of the program on fellows’ daily leadership experiences. Fellows received a skills logbook in which they documented the skills acquired from the didactic modules and the experiential attachment site period. Approval to publish routine fellowship program data was sought from the School of Medicine Research and Ethics Committee at MakCHS. All program activities, including but not limited to recruitment, module curriculum development, and module instruction and mentoring, were monitored by the International Training and Education Center for Health (I-TECH), a collaborative center operated jointly by the University of Washington and the University of California in San Francisco [4].

## Results and outcomes of fellows’ leadership-training experience

Between January 2011 and January 2015, out of 51 applications, 15 fellows (nine doctors and six nurses) participated in the program; four fellows annually except one intake when one fellow opted out after 3 months due to personal reasons. To date, all graduates of the program have remained in health leadership positions in Uganda. Overall, 13/15 (86%) of the fellows opted to take on new responsibilities as fellows within their employing institutions that were Afya Bora-accredited attachment sites. Annually, eight mentoring team meetings (including fellows, primary mentors, and attachment site mentors) were held during each fellowship. Activities at mentoring team meetings were tailored to fellows’ needs including technical support in research methodology, statistical data analysis, and peer review of scientific presentations and manuscripts. In addition, the team shared various mentoring experiences, challenges, opportunities, and the diverse projects at attachment sites. It cost the program up to $40 000 per fellow trained for a year. A majority (45%) of the funding was spent on fellows’ monthly stipend, and the remaining 55% was spent on module curriculum development and instruction, as well as travel for fellows and expert module facilitators to three blocks of 3-week residential modular training held in either of the consortium member countries (Botswana, Kenya, Tanzania, and Uganda) on a rotational basis.

### Attachment site projects

Overall, 14 (93%) of the 15 fellows conducted mentored projects, covering a wide spectrum of HIV/AIDS care and health system challenges, as highlighted in Table 1. One fellow opted for “only didactic modules,” an option that was provided only in the pilot year of the fellowship program. Subsequently, as an alumnus, she worked with another fellow on the family-planning project presented in Table 1. Matching fellows to projects that were mutually beneficial to the fellow and institution was considered extremely important to the success of the attachment site experience although it required time, in some cases up to 8 weeks. The main competences demonstrated by fellows included leading and motivating teams to produce results, strategic thinking, strategic planning, analysis of routine program data and its interpretation, and communication to inform the respective health service delivery programs, many of which were demonstrated through the various processes of execution of an attachment site project. Fellows completed a skills logbook (Additional file 1) to indicate all the skills acquired from both the didactic module and the attachment site period. With support from mentors, the program allowed fellows to dedicate up to 80% of their time to plan and implement these projects during a 9-month time frame. Results from specific projects were disseminated during meetings at the attachment sites, through presentations at national MOH stakeholders’ meetings, annual Afya Bora end-of-fellowship meeting, and other international meetings including International AIDS Society (IAS) conferences [7,8] and some were published in peer-reviewed journals [9-13]. Areas covered by fellows’ projects are described below.

**Table 1:** Attachment sites and spectrum of projects by Afya Bora global health leadership fellows in Uganda*

Attachment site	Type of site	Fellow’s project	Project outcomes
Ministry of Health—AIDS Control Program	Public (MOH)	HIV counselling and testing (HCT)	HCT policy review processes were analyzed and results widely disseminated. Recommendations were made to inform future policy formulation and review processes at MOH (Tumwesigye et al. Health Res Policy Syst. 2013).
		Policy review processes	
Ministry of Health—AIDS Control Program	Public (MOH)	Early infant diagnosis of HIV and the PMTCT cascade	Bottlenecks in the PMTCT cascade were identified including gaps in delivery of EID results to mothers/care takers of HIV-exposed children at local facilities. Recommendations were made to increase completion of PMTCT cascade for antenatal care to EID and enrollment into pediatric care (Elyanu P et al. IAS 2013).
Ministry of Health—AIDS Control Program	Public (MOH)	Early infant diagnosis of HIV and the PMTCT cascade	Predictors of perinatal HIV transmission in EID program at Jinja Regional Referral Hospital; presented at annual Afya Bora meeting and at the site. Results were used to improve follow-up systems for mothers and their HIV-exposed children after delivery at health centers served by Jinja Regional Referral Hospital.
Research Triangle International (RTI)	PEPFAR-funded HIV program	Implementation of PMTCT option B	Barriers to scale up of option B plus in a military setting in Bombo. Results were used to scale up uptake of PMTCT option B plus to mothers in the military HIV program at Bombo military barracks.
Ministry of Health—AIDS Control Program	Public (MOH)	Family-planning methods in HIV care programs	Utilization of birth control methods within HIV/AIDS care programs. Results were disseminated to the ministry of health to inform programs that scale up utilization of birth control methods (Muhindo et al., International Journal of Population Research, 2015).
Medical Research Council (MRC)/Uganda Virus Research institute	HIV/AIDS care program for risk populations	HIV risk in fishing communities	Access to HIV prevention methods for fishing communities in Uganda. Results were used to apply for a larger study to pilot use of HAART for prevention in high-risk fishing communities.
Infectious Diseases Institute (IDI), Makerere University College of Health Sciences (MakCHS)	Academic PEPFAR-funded HIV care outreach program at eight districts	Intra-facility linkage of HIV-infected mothers and HIV-exposed babies to long-term HIV care after PMTCT program	Analysis of routine PEPFAR HIV care program data to inform strategic implementation and health system strengthening (Mugasha et al. PLoS One 2014).
IDI, Makerere University	Male circumcision program		Factors influencing access to medical male circumcision (MMC) among IDI-supported clinics in Kampala. Results will be used to increase uptake on MMC.
Mbarara University for Science and Technology (MUST)	Academic/MOH referral hospital	Analysis of 10-year routine laboratory data for antibiotic resistance	Trends of antibiotic resistance were analyzed and disseminated widely to inform routine presumptive antibiotic prescriptions and essential drug lists at the MUST regional referral and teaching hospital (Bazira et al. British Microbiology Research Journal, 2014)
Strengthening Uganda’s Systems for Treating AIDS Nationally (*SUSTAIN*), Uganda	PEPFAR at 19 MOH AIDS care sites in Uganda	Outcomes of PMTCT program at regional referral hospitals in Uganda	Breastfeeding practices and antiretroviral therapy (ART) predicted outcome of PMTCT. Scale up of ART and DNA PCR were recommended (presented at the national pediatric HIV conference, Uganda, 2014).
Infectious Diseases Institute (IDI) training department	Academic regional center for HIV/AIDS care training in Africa	Continuing health professionals’ education	Provision of continuing health professionals’ education in HIV/AIDS care at IDI; was presented at the annual Afya Bora end-of-fellowship meeting. Results were used to develop other curricula in the department of nursing, Makerere University
Infectious Diseases Institute (IDI) training department	Academic regional center for HIV/AIDS care training in Africa	Monitoring and evaluation of PMTCT training module	Knowledge, competence, and experiences of health workers in Kampala City Council Authority (KCCA) facilities 1 year following PMTCT option-B plus training for health workers. Results were used to modify the PMTCT training module to include more practical sessions.
Makerere University College of Health Sciences	Academic institution, nursing department	Ethics training for nurses	Nurses’ knowledge in ethics and their perceptions regarding continuing ethics education; a cross-sectional survey among nurses at referral hospitals in Uganda. Results will be used to improve pre-service and in-service ethics training for nurses (Osingada et al. BMC Res Notes. 2015)
Makerere University College of Health Sciences	Academic institution, nursing department	Psychosocial adaptation during antiretroviral therapy	Psychosocial adaptation and antiretroviral therapy (ART) adherence of HIV+ adults in Kampala, Uganda. Results will be used to improve adherence counselling for patients receiving life-long ART at IDI and other HIV-treatment programs

*One fellow did modules only and did not participate in an independent project. During the planning and execution of these projects, fellows were mentored to develop a wide spectrum of competences including but not limited to leading and motivating teams to produce results, strategic thinking, strategic planning, and analysis of routine program data and its interpretation, as well as oral and written communication to inform practice

### Use of routine national HIV program data

National HIV program data were used to identify gaps and guide interventions to improve pediatric HIV/AIDS care (Table 1). In order to address gaps identified during the fellowship period in the continuum of national Prevention of Maternal to Child Transmission of HIV (PMTCT) and early infant diagnosis (EID) of HIV programs [7], fellows developed highly competitive proposals, and 4/15 (27%) fellows received career development awards to improve early infant diagnosis of HIV among HIV-exposed babies and disclosure of HIV-sero-status to adolescents born with HIV to promote adherence to long-term HIV/AIDS care programs (Table 2). In addition, gaps in intra-facility linkage of mothers and babies to chronic HIV care after antenatal PMTCT and EID programs were identified. In response to the results, integration of HIV care and EID has been implemented at Infectious Diseases Institute (IDI)-supported HIV/AIDS care facilities to reduce attrition of HIV-infected mothers and HIV-exposed babies [10].

**Table 2:** Effect of Afya Bora fellows’ activities on their capacity to support improvement of health care in Uganda

Thematic areas assessed	Reponses from the fellows
New responsibilities assigned to fellow due to skills acquired during the fellowship	“I am involved in grant application writing for different projects, a role that was previously left to scientists more senior than me.”
	“I participate in peer review of journal articles.”
	“I am now participating in multi-disciplinary research projects.”
	“I am currently the head of department of Microbiology and also the coordinator of graduate programs in the faculty of medicine at Mbarara University of Science and Technology.”
	“Following training experience I was given additional responsibilities from technical advisor to project manager role for elimination of mother to child transmission of HIV (eMTCT) programs.”
	“Immediately after Afya Bora fellowship, I was hired at the AIDS information Center, with a major role of ensuring integration of family planning services into HIV/AIDS services.”
	“The leadership and communication skills acquired during Afya Bora helped me to compete successfully for the position of director of a regional referral hospital.”
Improvements in quality of work	“Skills acquired in Afya Bora fellowship have helped me to improve our grant applications and to refine study objectives and research strategies for different study protocols.” “I have applied some of the study methodologies I learnt during the epidemiology course to new studies at MRC/UVRI.”
	“I have used the knowledge on manuscript writing to publish over 10 papers ever since I completed the fellowship.”
	“I have also participated in writing a training grant that is now under review.”
	“I have functionalized the use of quality improvement across thematic areas of pediatric HIV, TB/HIV, eMTCT and linkage to HIV care services.”
	“I have also been able to advocate for more practical and problem-based approaches for capacity building training curriculum with MoH and other implementing partners.”
	“I have supported development and mentorship of human resource at IDI especially for those that I supervise through performance-driven appraisal system.”
	“I have used acquired skills in monitoring and evaluation to improve the hospital outputs more especially the newborn care outcomes.”
New projects/innovations/changes you have developed in your program after the Afya Bora fellowship	“I have participated in grant application for two studies: one on non- communicable diseases and another on pre-exposure prophylaxis.”
	“I have led the start of grand rounds/seminars for my whole faculty of medicine.”
	“I have modified the quality improvement documentation journal to help health workers review performance.”
	“We have integrated family planning and cancer cervix screening in our PMTCT program.”
	“I am working on integration of adolescent HIV care within the clinics.”
	“I have linked private clinics in 4 districts (Mpigi, Mayuge, Mityana, Kumi, kaliro) to the national contraceptive supply system.”
	“I have introduced surveillance of newborn care at Jinja regional referral Hospital.”
Dissemination conferences attended	“I have presented my work at 1 local and 4 international scientific meetings, 4 peer-reviewed publications and 4 manuscripts in development.”
Other ways in which your fellowship experience has improved health care delivery	“Through the fellowship training I improved my data quality management skills for both qualitative and quantitative data collection. This helps to ensure that we generate quality data which is necessary for healthcare planning and delivery.”
	“Through improved communication skills and implementation science I have supported the programs to meet and respond to emerging needs even as we implement. I have also supported proposal writing for 2 projects IDI has won to provide additional funding within districts we support.”
	“I am working with two other Afya Bora fellows to develop a project on provision of family planning services to unreached communities in the mountains of South western Uganda.”
	“The program has improved my communication skills.”

*PMTCT* prevention of mother-to-child transmission of HIV, *IDI* Infectious Diseases Institute, *eMTCT* elimination of mother-to-child transmission of HIV

### Women’s uptake and adherence to family-planning services

As a result of a project undertaken to understand predictors of women’s adherence to family-planning methods [11], carried out during the fellowship, a fellow was employed by the AIDS Information Center (AIC) in Uganda to work on integration of family-planning services within the HIV/AIDS care program. The fellow has subsequently linked private clinics in five districts (Mpigi, Mayuge, Mityana, Kumi, Kaliro) to the national contraceptive supply system, under a private sector mechanism (Table 2).

### Analysis of routine data to guide strategic innovations to improve health care delivery

Analysis of 10-year laboratory data was conducted, for the first time, by an Afya Bora fellow. It was reported that *Staphylococcus aureus* isolates had up to 90% resistance to commonly prescribed oral antibiotics such as amoxicillin, cloxacillin, and erythromycin. Regular review of antibiotic-resistance patterns was initiated to guide updates of the hospitals’ essential drug lists and empirical antibiotic prescriptions, where required [9].

### Documentation and review of policy formulation and review processes at the national AIDS control program

The fellowship equipped fellows to lead an internal review of the policy formulation and review processes to challenge the status quo and become change agents. These results guided the development and implementation of standard operating procedures as well as increased involvement of the policy review unit in subsequent policy formulation and review processes [12].

### Evaluation of training curricula and their effect on health care programs

At the IDI, Makerere University, a regional center of excellence in training, research, and HIV care in Africa, a fellow reviewed challenges faced by the training program for HIV/AIDS care providers in sub-Saharan Africa. The two common concerns identified about the IDI model of educational/training delivery were (a) the fact that health workers move to IDI for the training which creates a gap in service delivery within the already under-staffed health facilities and (b) the high cost of residential training programs that limits sustainability. These results have guided the development of HIV/AIDS care distance-learning modules to train health workers at minimal costs and with minimal disruption of service delivery.

### Post-fellowship activities

Recommendations from fellows’ work were used to develop evidence-based interventional projects that were funded by the Afya Bora career development awards ($40 000 each). Post-fellowship career development awards were won by fellows to continue innovative projects based on results from small projects conducted during the fellowship (Table 3).

**Table 3:** Post Afya Bora fellowship career development awards to improve HIV/AIDS care delivery in Uganda

Institution	Post Afya Bora career development award project title
Makerere University Infectious Diseases Institute	Disclosure of HIV-sero-status among adolescents living with HIV
Ministry of Health AIDS Control Program	Factors associated with failure to return for infants’ HIV test results in Uganda
Ministry of Health AIDS Control Program	Improving uptake into psychosocial support structures for HIV-infected pregnant women at public hospitals in Uganda: a randomized trial of a psychosocial assessment guide intervention
Medical research Council/Uganda Virus Research Institute	Acceptability of PrEP and factors likely to influence its uptake among fisherfolk in Uganda: a discrete choice experiment

### Post-fellowship leadership roles of Afya Bora fellowship alumni

Upon completion of the 1-year global health leadership program (consisting of a total of 9 months of attachment site experience and a total of 3 months of didactic modules), fellows were empowered to take on more leadership roles; for example, one fellow was appointed director of a regional referral hospital and another fellow was appointed coordinator of the Early Infant Diagnosis program at the national Ministry of Health AIDS Control Program. In addition, fellows have highlighted individual and institutional benefits from the skills gained during the program and how acquired skills were used to take on new responsibilities and use innovative approaches to improve health care delivery programs within their respective settings (Table 3).

## Discussion

An important fellowship objective was to mentor clinician fellows to help them develop critical-thinking skills and implement projects that would optimize health service delivery programs at the attachment sites. The Afya Bora leadership-training program therefore provided fellows opportunities to select projects of interest to the fellows and to the attachment sites’ agenda to improve health care [4]. Having the fellows as leaders of their own attachment site experience required a paradigm shift from most in-service training programs that detach trainees from a work environment in a search for protected time. Mentorship by attachment site mentors from the respective sites and primary mentors from the Afya Bora program was critical to development of locally relevant projects.

Potential projects usually did not require extra funding but rather required protected time to generate and use evidence from existing data sets and activities at the attachment site institutions. This was in line with previous reports from India that building the leadership capacity of health support workers, doctors, and managers increased the systems’ capacity for more effective use of resources including time [6]. The Afya Bora program complements other efforts by international consortia such as the Academic Alliance for AIDS Care and Prevention in Africa (AA), initiated by 14 university-based Ugandan and North American physicians in 2001, to build capacity in training, research, prevention, and care of HIV/AIDS and other infectious diseases [14]. Through the AA initiative, the Infectious Diseases Institute (IDI) at Makerere University has supported masters-level-, doctoral-, and post-doctoral-training programs to provide a strong research foundation for individuals to perform independent, original research to answer locally relevant questions [15]. Similarly, the National Institute of Health (NIH)-funded Medical Education Partnership Initiative (MEPI) has contributed to increase the number of trained health care providers, improve quality of medical education, and strengthen evidence-based health workforce planning to improve health care in resource-limited settings [16,17]. It is, however, important to consider that research uptake/utilization and its effect remain largely dependent on individual leadership capacity and governance to influence decision making and improve health system performance [3,6]. Given that many health facility leaders in Uganda do not have mandatory management and leadership training [6], there is a need for in-service training in health leadership and governance to equip health leaders with knowledge and practical skills necessary to negotiate and influence in-country health care decisions. The Afya Bora program therefore is appropriate to equip health workers with leadership skills required to influence the translation of research to policy through strategic communication of health care problems to policy makers and to provide fellows with monitoring and evaluation skills and data utilization skills to influence practice.

To promote sustainability of the health leadership training in Africa, curriculum development and training modules were co-led by African and American trainers. In addition, alumni from the program are usually invited to participate in program activities as co-facilitators, interviewers, and mentors of subsequent fellows, to further build local capacity to run the program. The authors postulate that use of developed curricula and local trainers will decrease travel requirements and cost of delivery of the 1-year leadership training beyond the Afya Bora program period. Country leads and graduates of the Afya Bora fellowship program are expected to spearhead the scale up of in-service leadership training for health care providers and advocate for integration of essential leadership competences into medical-training curricula in sub-Saharan Africa. Developers of the Afya Bora program are happy to provide copies of modules and training materials for individuals in other countries who may be interested in replicating the global health leadership program.

A previous review of health performance in Uganda revealed a heavy reliance on external technical assistance to undertake major roles that local institutions would need to ensure sustainable high-quality performance in the health sector [3]. The authors believe, and as demonstrated by the fellows, that practical leadership-training models like the Afya Bora program equip doctors and nurses with the practical leadership skills required to champion great improvements in health care delivery.

All fellows (alumni of the first 4 years of the program) have remained in Uganda. The authors postulate that innovative Afro-centric programs, such as Afya Bora, may reduce the movement of trained health professionals from Africa to other nations to seek better career opportunities [18]. We recommend long-term follow-up of Afya Bora trainees, for example, through alumni career tracking, to monitor how fellows continue to use the leadership skills acquired to improve health care beyond the fellowship. The authors also recommend cost-effectiveness analysis of delivery of this 1-year leadership-training fellowship in resource-limited settings. Furthermore, we recommend a critical analysis of similarities and disparities in the uptake of the Afya Bora program in the other African member countries of the consortium.

### Lesson learned

Our program activities were in line with the people-centered framework for health system strengthening, which emphasizes that skilled leaders influence health care providers, clients, and government systems to improve performance, access, and use of high-quality health care services to improve health outcomes [5].

We demonstrated that 10 months of mentored practical leadership training for clinical fellows (nurses and doctors) within their employment stations (at academic and local health care programs) contributed to increased individual and institutional capacity for critical analysis of gaps in health care systems.

It was feasible to have in-service leadership training through identification of protected time for fellows to generate evidence-based solutions to challenges within their work environment.

Matching fellows to projects that were mutually beneficial to the fellow and institution required up to 8 weeks in some cases, and fellows needed the support from mentors at the respective attachment sites as well as primary mentors from the Afya Bora program.

Joint mentorship, by primary mentors (from MakCHS) and attachment site mentors (from MOH, NGOs, and national PEPFAR-funded initiatives), provided fellows with a good mix of skill sets that is essential for global health leadership.

There was a wide spectrum of potential projects in which fellows were involved including data analysis and dissemination and use of routine program data to guide practice and policies to improve health care delivery at academic, public, and non-governmental health care programs in Uganda.

Tracking of alumni is required to determine how fellows continue to use the leadership skills they acquired to influence health care programs.

Co-facilitation of the didactic modules by African and American trainers helped to the build capacity of African leaders to train fellows using curricula that were jointly developed by working group members from the eight consortium universities.

Leadership training is critical to maximize the benefits of medical training. There is need to consider strengthening our formal medical-training program with components of leadership skills that are much needed to improve health service delivery.

The Afya Bora leadership program was limited to nurses and doctors. In the future, this could be expanded to include other cadres that support health care delivery including laboratory staff, social workers, health administrators, and policy makers, in cases where the latter are not clinicians.

Strategic investment in practical global health leadership training, at academic institutions in collaboration with public HIV/AIDS care programs, should be developed to equip nurses and doctors to make evidenced-based decisions to address challenges to health care in Africa.

## Conclusion

In-service leadership training was feasible, with ensured protected time for fellows to generate evidence-based solutions to challenges within their work environment. With structured mentorship, collaborative activities at academic institutions and local health care programs equipped health care providers with leadership skills. Inputs from the fellows’ increased ability to influence the performance of health care delivery systems should contribute to improved health outcomes in resource-limited settings.

## Additional file

Additional file 1:
**Afya Bora fellowship skills log book.** Fellows completed the skills logbook to indicate all the skills acquired from both the didactic modules and the attachment site period.
