# Supplementary material for: Global health leadership training in resource-limited settings: a collaborative approach by academic institutions and local health care programs in Uganda
Source: Hum Resour Health. 2015 Nov 18;13:87. doi: 10.1186/s12960-015-0087-2 (PMC4650924; doi:10.1186/s12960-015-0087-2)
Supplement: Additional file 1: — Afya Bora fellowship skills log book. Fellows completed the skills logbook to indicate all the skills acquired from both the didactic modules and the attachment site period. [file 12960_2015_87_MOESM1_ESM.docx]

| **Core Skill** | **Description of how demonstrated** | **Mentor Signature** |
| --- | --- | --- |
| **Communications Skills** | | |
| Effectively conveyed difficult information to a client, team member or stakeholder using SPIKES |  |  |
| Developed an executive summary to convey information or marketing strategy |  |  |
| Wrote and/or published a scientific manuscript |  |  |
| Prepared and/or prepared an elevator speech to a group |  |  |
| Created a professional CV |  |  |
| **Health Informatics** | | |
| Evaluated or utilized one of the following technologies to collect or manage health information and data: EpiInfo, openMRS, Magpi (Datadyne), Open Data Kit, Google docs, Dropbox |  |  |
| Developed, selected, and/or implemented a health data management system |  |  |
| Developed a strategy to improve quality of health data collection and management |  |  |
| Utilized a health surveillance system to analyze disease trends |  |  |
| Trained colleagues on health data collection strategies |  |  |
| **Implementation Science** | | |
| Considered experimental and quasi-experimental designs to generate evidence on the impact of health programs |  |  |
| Identified which economic analysis approaches are most appropriate to answer specific policy and implementation related questions |  |  |
| Applied systems analysis and improvement techniques to strengthen health programs and delivery approaches within their workplace context |  |  |
| Identified and mapped stakeholders to support development of evidence-based health programs and related policies |  |  |
| Designed an information dissemination (and, if appropriate) marketing approach for a health program |  |  |
| **Leadership Skills** | | |
| Critically thought and set priorities for programmatic goals |  |  |
| Critically analyzed a problem and developed a coherent strategic plan to address it |  |  |
| Built a consensus among disparate parties using mediation or other techniques |  |  |
| Organized logistics and overcame obstacles to implement a program |  |  |
| Demonstrated initiative and/or innovation in a problem-solving situation |  |  |
| **Monitoring and Evaluation** | | |
| Used a logic model tool as a framework for expected program inputs, outputs, outcomes and impacts |  |  |
| Selected and customized indicators that can be used for program-specific questions |  |  |
| Developed a monitoring and evaluation plan |  |  |
| Developed and/or implemented a data collection plan |  |  |
| Analyzed, synthesized, and/or communicated data using descriptive and statistical methods |  |  |
| **Responsible Conduct of Research** | | |
| Considered own conflict of interest and completed a conflict of interest form |  |  |
| Completed a human subjects section of a grant or research proposal |  |  |
| Participated in or contributed to a peer review process for a manuscript or grant |  |  |
| Participated in or contributed to a review process for a research protocol |  |  |
| Designed or oversaw systems to ensure confidentiality in data collection, storage, or management. |  |  |
| **Research Methods** | | |
| Used quantitative methods to understand a problem/question |  |  |
| Created a box plot, bar graph, or other graph |  |  |
| Applied any of the following statistical concepts to quantitative data sets: causation, bias, confounding, classification, and misclassification |  |  |
| Utilized qualitative methods to understand a problem/question |  |  |
| Developed qualitative research tools including any of the following: interviews, videos, observations, focus groups, case report forms, surveys, etc. |  |  |
| **Human Resources and Budgeting** | | |
| Evaluated an employee’s performance |  |  |
| Completed job analysis |  |  |
| Designed and/or facilitated training events |  |  |
| Designed and/implemented retention strategies |  |  |
| Developed a budget using excel, and/or presented it in Power point. Justification was provided. |  |  |
| Developed a system of monitoring and tracking budgets |  |  |
| Implemented a budget or parts of a budget |  |  |
| **Grant writing** | | |
| Reviewed and selected potential funding sources for a grant |  |  |
| Wrote and finalized specific aims for a grant |  |  |
| Identified domains of knowledge relevant to grant application |  |  |
| Selected a study design and described methods, eligibility, criteria, and study procedures |  |  |
| Completed and submitted a grant application |  |  |
| **Project management** | | |
| Created a project design |  |  |
| Designed and utilized any of the following frameworks: Gantt chart, work break down structure, PERT chart |  |  |
| Implemented the project design or segments of the project design |  |  |
| Evaluated project risks |  |  |
| Reviewed the project’s outcomes |  |  |
| **Global health policy and governance** | | |
| Utilized global health policy concepts to analyze issues in current employment or attachment site context |  |  |
| Identified and/or contacted potential donor organizations |  |  |
| Identified barriers to working across organizations, for example, competing interests of donor and recipient organizations. |  |  |
| Identified organizations that can provide assistance in the development of health policy. This can include legal organizations, global health organizations. |  |  |
| Completed a stakeholder analysis |  |  |
